# Cross-National Generalizability of WISC-V and CHC Broad Ability Constructs across France, Spain, and the US

**DOI:** 10.3390/jintelligence11080159

**Published:** 2023-08-07

**Authors:** Christopher J. Wilson, Stephen C. Bowden, Linda K. Byrne, Louis-Charles Vannier, Ana Hernandez, Lawrence G. Weiss

**Affiliations:** 1 Melbourne School of Psychological Sciences, The University of Melbourne, Melbourne 3010, Australia; 2 Research and Development, Pearson Clinical Assessment, Melbourne 3121, Australia; 3 Department of Clinical Neuroscience, St. Vincent’s Hospital, Melbourne 3065, Australia; 4 Faculty of Psychology, Counselling & Psychotherapy, The Cairnmillar Institute, Melbourne 3123, Australia; 5 Psychometrics, Pearson Clinical Assessment, 75012 Paris, France; 6 Research and Development, Pearson Clinical Assessment, 08011 Barcelona, Spain; 7 Test Development Consultant, Satellite Beach, FL 32937, USA

**Keywords:** Wechsler Intelligence Scale for Children-Fifth Edition, measurement invariance, confirmatory factor analysis, construct validity, intelligence

## Abstract

The Cattell–Horn–Carroll (CHC) model is based on psychometric cognitive ability research and is the most empirically supported model of cognitive ability constructs. This study is one in a series of cross-national comparisons investigating the equivalence and generalizability of psychological constructs which align with the CHC model. Previous research exploring the cross-cultural generalizability of cognitive ability measures concluded that the factor analytic models of cognitive abilities generalize across cultures and are compatible with well-established CHC constructs. The equivalence of the psychological constructs, as measured by the Wechsler Intelligence Scale for Children-Fifth Edition (WISC-V), has been established across English-speaking samples. However, few studies have explored the equivalence of psychological constructs across non-English speaking, nationally representative samples. This study explored the equivalence of the WISC-V five-factor model across standardization samples from France, Spain, and the US. The five-factor scoring model demonstrated excellent fit across the three samples independently. Factorial invariance was investigated and the results demonstrated strict factorial invariance across France, Spain, and the US. The results provide further support for the generalizability of CHC constructs across Western cultural populations that speak different languages and support the continued use and development of the CHC model as a common nomenclature and blueprint for cognitive ability researchers and test developers. Suggestions for future research on the CHC model of intelligence are discussed.

## 1. Introduction 

Generalizing the measurement of psychological constructs across populations requires the demonstration of measurement invariance (AERA et al. 2014; ITC 2017). Further, this demonstration allows for the generalizability of construct validity in terms of convergent and discriminant validity (Jewsbury and Bowden 2017). Construct validity can be defined as how well a set of tests, or an assessment battery, accurately measures the constructs of interest (e.g., working memory, fluid reasoning etc.). Establishing construct validity thus provides evidence that the same construct is evident across different populations and that the construct can be accurately measured and compared. However, psychological constructs are not directly measurable (i.e., latent) but are estimated using various techniques, including confirmatory factor analysis (CFA). In this way, a factor model provides a description of the statistical and theoretical relationship between observed test scores and the corresponding latent variables or constructs.

Cognitive ability assessment is founded on psychometric theory and factor analysis. Spearman (1904) initially theorized that a single factor ‘g’ (or general intelligence, or Spearman’s ‘g’) would explain the intercorrelations across intelligence tests. Thurstone (1938) later proposed that many factors account for the variance across intelligence tests. The theory of cognitive ability later grew with the work of Cattell (1943, 1963), who suggested that ‘g’ could be divided into two methods of problem-solving, a ‘fluid’ and ‘crystallized’ intelligence (Gf-Gc Theory). This theory was then developed by the work of his student, Horn (1986), who expanded the model and suggested there are multiple constructs of intelligence. However, it was the seminal work of Carroll (1993) and the factor analysis of over 450 data sets that led to a hierarchical model of cognitive abilities. From this work emerged what is a widely accepted and empirically supported model of intelligence, the Cattell–Horn–Carroll (CHC) model (McGrew 2009; Schneider and McGrew 2018). Carroll’s factor analytic research demonstrated that a vast array of cognitive ability tests are not unidimensional but, instead, comprise a finite set of positively correlated (positive manifold) latent abilities (Schneider and Newman 2015). The CHC framework describes a three-stratum model of intelligence, with general intelligence at the top, broad abilities (first-order factors) at the second stratum, and narrow, mostly tests-specific abilities, at stratum one. It is the first-order factors, or broad abilities under the CHC framework, which are identified by CFA and are of most importance in terms of construct validity (Jewsbury and Bowden 2017). For a comprehensive explanation and review of the current literature on the CHC model, see Schneider and McGrew (2018).

As a test of construct validity, measurement invariance analysis uses CFA across multiple groups. Evaluation of the invariance of the factor structure across groups is described as ‘factorial invariance’ (Meredith 1993; Widaman and Olivera-Aguilar 2023). If established, factorial invariance implies that test scores and latent factors (constructs) are generalizable across groups (Horn and McArdle 1992). Thus, establishing factorial invariance is necessary to allow for the comparison of constructs across groups and allows for the meaningful comparison of latent mean scores (Widaman and Reise 1997). Factorial invariance also provides evidence that psychological constructs, which align with the broad abilities in the CHC model, are present across diverse populations.

Factorial invariance analysis is conducted in an increasingly restrictive hierarchical approach first described by Widaman and Reise (1997), (cf. Bontempo and Hofer 2007; Meredith and Teresi 2006; Vandenberg and Lance 2000). First, configural invariance requires only that the same indicator–factor pattern displays good fit across groups and, if found, provides evidence that the psychological constructs and organization of items to constructs are the same across groups (Horn and McArdle 1992). Second, weak factorial invariance adds the constraint of the equality of factor loadings and, if established, suggests that the unit of measurement does not differ across groups, thus allowing for the generalization of construct validity interpretations (Widaman and Reise 1997). Third, strong factorial invariance adds the constraint of equality of intercepts across groups and, if found, permits a meaningful comparison of latent factor mean scores across groups (Meredith 1993). Lastly, strict factorial invariance adds the constraint of equality of residuals across groups and, if established, implies that the common factors are the cause of any group differences in means and variances (Widaman and Reise 1997).

The Wechsler Intelligence Scale for Children-Fifth Edition (WISC-V) is the latest edition of the most widely used assessment of intelligence for children aged 6–16 in the world (Kaufman et al. 2016). The current fifth edition of the WISC consists of 15 subtests in the French and Spanish versions or 16 subtests in the US factor analytic version and, in every national version, measures five factors or primary indexes of intelligence which closely align with broad cognitive abilities in the CHC framework (Reynolds and Keith 2017; Schneider and McGrew 2018). See Table 1 for definitions of the WISC-V factors and corresponding CHC broad abilities.

The current study is one in a series of studies of cross-national comparisons investigating the equivalence and generalizability of CHC psychological constructs using the WISC-V. First, a systematic review exploring the cross-cultural generalizability of cognitive ability measures concluded that the factor analytic models of cognitive ability assessments consistently generalize across cultures and that the factor analytic models of intelligence assessments are compatible with the CHC constructs (Wilson et al. 2023b). Second, the equivalence of the psychological constructs, as measured by the WISC-V, was established via strict metric factorial invariance, initially across Australia and New Zealand (A&NZ) and the US (Wilson et al. 2023c) and then across the UK, A&NZ, and the US (Wilson et al. 2023a). These cross-national English-speaking factorial invariance results provide strong evidence that the CHC-compatible constructs, as measured by the WISC-V, can be generalized across A&NZ, the UK, and the US. However, few studies have explored the equivalence of psychological constructs across non-English speaking nationally representative samples.

The measurement invariance of a five-factor CHC-aligned model using the WISC-IV was investigated across the French standardization sample and a stratified French-speaking Swiss sample (Reverte et al. 2015). While weak factorial invariance was established, suggesting the CHC factors have the same meaning across samples, strong invariance was not demonstrated, possibly because of the relatively modest sample size of the French-speaking Swiss sample.

Further, factorial invariance was examined across English and Italian standardization samples using the cognitive assessment system (CAS), which is based on the PASS (planning, attention, simultaneous, and successive) theory of intelligence (Naglieri et al. 2013). The results supported strict factorial invariance across samples based on the guidelines of interpreting adequate model fit of the root mean error of approximation (RMSEA) <.08 (Browne and Cudeck 1993). However, the findings of a joint CFA of the CAS and the CHC broad abilities, as measured by the Woodcock-Johnson Test of Cognitive Abilities-3rd edition, did not support the construct validity of the CAS as a measure of the PASS theory of individual difference in intelligence but, rather, it has been suggested that the PASS model may have significant overlap with CHC constructs (Keith et al. 2001; Schneider and McGrew 2018).

Additionally, the factorial invariance of the three-factor structure of the Wechsler Memory Scale-Revised was tested across standardization data sets from the US and Japan (Omura and Sugishita 2004). The results supported the configural invariance of the three memory dimensions (attention/concentration, immediate memory, and delayed recall), providing evidence for the generalizability of the memory constructs; however, weak factorial invariance was rejected.

Recently, the factorial invariance of cognitive abilities was explored using translated versions of the NIH Toolbox Cognitive battery (NIHTB-CB) across adult community samples of three low- and middle-income countries; Guatemala, the Philippines, and South Africa (Wray et al. 2020). While configural invariance was concluded, weak invariance could not be established, suggesting the unit of measurement of the factors was different across samples.

Lastly, the NIHTB-BC was used to investigate the factorial invariance of English-speaking and Spanish-speaking adults taken from the normative sample data set (Karr et al. 2022). Strict invariance was found across languages for a two-factor model (labelled crystallized cognition and fluid cognition) allowing for the direct comparison of latent means across groups. However, both samples were recruited and assessed within the United States, restricting the cross-cultural generalizability of the findings.

The present study aimed to investigate the generalizability of the CHC broad ability constructs, as measured by the WISC-V, across French, Spanish, and US nationally representative samples. Based on the previous research investigating the factorial invariance of the WISC-V across countries (see van de Vijver et al. 2019; Wilson et al. 2023a, 2023c), it was hypothesized that the WISC-V would display factorial invariance (i.e., weak, strong, or strict factorial invariance) across French and US, Spanish and US, and French and Spanish normative samples. Establishing factorial invariance across three different language-speaking nationally representative samples would provide further support for the cross-national and cross-cultural generalizability of the CHC model. The implications of the construct validity of the WISC-V test scores across France, Spain, and the US, and the WISC-V factor compatibility with Carroll’s three-stratum theory, will be discussed.

## 2. Methods

### 2.1. Participants

This study used three nationally representative standardization samples from the normative data projects of the WISC-V France, WISC-V Spain, and WISC-V US. The French sample included 1049 participants, stratified by parental level of education, gender, age, and region, matched to the French census. The Spanish sample included 1008 children, stratified by parental level of education, gender, age, and region, matched to the Spanish census. The US sample was 2200 children representative of the US English-speaking population by age, gender, race, parental education level, and geographic region, according to the US census. All participants across the three samples were aged 6–16 and were divided evenly into 11 age groups by year of age. See Table 2 for demographic information on these nationally representative samples. Previous measurement invariance CFA analysis on the WISC-V Australia and New Zealand determined that samples over 500 provide a beta-power greater than the commonly accepted 0.80, alpha equal to 0.05, to achieve minimal bias and adequate statistical power to identify a multifactorial structure (Wilson et al. 2023c).

### 2.2. Procedure

Participants in the French sample were administered all 15 subtests as part of the French WISC-V standardization. French data collection ran from 2014 to 2015. All examiners were experienced and trained psychologists. For further information on the test development and test procedures, see the WISC-V French Manual (Wechsler 2015b). Participants in the Spanish sample were administered all 15 subtests as part of the Spanish WISC-V normative development. Data collection for the standardization of the Spanish WISC-V ran from 2014 to 2015. All examiners were trained and registered psychologists. Additional information on the development of the Spanish WISC-V and study design can be found in the WISC-V Spanish Manual (Wechsler 2015a). Lastly, the US standardization sample participants were administered all 21 subtests of the WISC-V US normative development. Six of the 21 subtests from the US WISC-V were not included as part of published versions of the French and Spanish WISC-V (picture concepts, naming speed literacy, naming speed quantity, immediate symbol translation, delayed symbol translations, and recognition symbol translation) and were thus excluded from all analyses. Data collection for the normative development of the US WISC-V ran from 2013 to 2014. See the technical and interpretative manual for full details on the development of the US WISC-V (Wechsler 2014).

### 2.3. Analysis

Baseline estimation:

Subtest raw score data for the three samples were used for all analyses as any invariance found in the raw scores will also apply to any transformed scores, such as scaled or index scores (Widaman and Reise 1997). The use of raw score data instead of scaled score data may result in higher factor correlations due to the extended range of scores (Bowden et al. 2007). Data were cleaned and 22 cases from the French sample, seven cases from the Spanish, and three cases from the US sample were removed because of missing data in one or more subtests. Confirmatory factor analysis (CFA) using Mplus 8.5 (Muthén and Muthén 2020) was first undertaken to establish the best fitting model in the French, Spanish, and US normative samples independently to serve as a baseline model for further tests of measurement invariance. CFA was used in preference to exploratory methods as CFA has been shown to provide less biased estimates of the factor correlations (Little et al. 1999). Maximum likelihood estimation was used as it is robust to minor departures in normality (Brown 2015). All CFA models were identified by fixing the loading of the first subtest (indicator) in each factor to unity by default to become the marker indicator.

A one-factor model, where all 15 subtests load onto a single factor (analogous to Spearman’s *g*), was investigated first in each sample to serve as a simple baseline comparison for further, more complex models (Agelink van Rentergem et al. 2020; Kline 2016). A previously published four-factor model using the WISC-IV was next examined for each sample and the model fit was compared to the simple one-factor model. The four-factor model comprised of a (i) verbal comprehension (Gc) factor which loaded onto similarities, vocabulary, information, and comprehension; (ii) a perceptual organization (Gv and Gf composite) factor which loaded onto block design, visual puzzles, matrix reasoning, and figure weights; (iii) a working memory (Gwm) factor which loaded on to arithmetic, digit span, picture span, and letter number sequencing; and (iv) a processing speed factor (Gs) which loaded on coding, symbol search, and cancellation (Schneider and McGrew 2018; Sudarshan et al. 2016; Weiss et al. 2013).

Next, the five-factor scoring model of the WISC-V, published in the respective manuals for France and Spain, was investigated in the French, Spanish, and US samples, comprising verbal comprehension (VC or Gc), visual spatial (VS or Gv), fluid reasoning (FR or Gf), working memory (WM or Gwm), and processing speed (PS or Gs) factors (see Table 1 for CHC broad abilities to WISC-V factors correspondence).

To determine the best fitting baseline model for further tests of measurement invariance, the chi-square test was reported; however, the test has been shown to be overly sensitive in large samples, so the emphasis was placed on the alternative fit indices, namely, root mean error of approximation (RMSEA), comparative fit index (CFI), Tucker–Lewis index (TLI), standardized root mean square residual (SRMR), gamma hat, Akaike information criterion (AIC), and Bayesian information criterion (BIC), in line with current recommendations (Brown 2015; Cheung and Rensvold 2002; Marsh et al. 2004; Meade et al. 2008). Like chi-square, AIC, BIC, SRMR and gamma hat (a modified version of the goodness-of-fit index) indices, are absolute fit indices that do not use an alternative model as a base for comparison (Hu and Bentler 1999). Good fit of the baseline model was supported by an SRMR value below 0.080, an RMSEA below 0.060, and CFI, TLI, and gamma hat values greater than 0.950 (Brown 2015; Hu and Bentler 1999). In regard to AIC and BIC fit indices, the models with the lowest values were considered to fit the data better compared to the other models (Brown 2015). The difference in chi-square was reported to test if the more complex factor solution showed significantly better fit compared to the less complex nested model (Gorsuch 2003).

When determining the baseline model for further tests of measurement invariance, second-order models were not reported due to the statistical limitations of second-order model identification. A model must be identified in order for the unknown parameters in the model to be estimated. Underidentification, where there are more parameters than correlations, can lead to improper solutions such as standardised values over 1.0 or negative error variances, sometimes called Heywood cases (Bentler and Chou 1988; Chen et al. 2001). Negative error variances are impossible values in the population and are a symptom of structural misspecification (Kolenikov and Bollen 2012). While model identification is a common problem in factor analysis, no sufficient conditions of model identification are known (Bollen and Davis 2009). However, a distinction exists between algebraic underidentification and empirical underidentification. Empirical underidentification occurs when, in principle, the system of equations may be algebraically identified (i.e., positive degrees of freedom); however, in practice, there is no solution for a parameter due to insufficient covariance information (Kenny 1979; Kenny and Milan 2012; Rindskopf 1984). Empirical underidentification in factor analysis can occur if a factor loading approaches zero, the correlations between two factors is high (e.g., higher than 0.9), or a model is specified with a factor with only two indicators in a larger model, such as higher order models (Bentler and Chou 1988; Kenny 1979; Rindskopf 1984). While empirical underidentification can be difficult to identify (Bentler and Chou 1988), it is suggested that when the analytic software “declares a model unidentified that is algebraically identified, the most likely cause is empirical under-identification” (Rindskopf 1984, p. 117). Further, the software may produce statistically impossible population estimates such as negative variances or correlations greater than one (Kenny and Milan 2012). Encountering errors in software outputs, such as negative variances, should not be ignored and reflects an improper solution, and is usually a consequence of poor model identification (Newsom et al. 2023). When negative estimates of error variances occur, researchers are encouraged to screen for empirical underidentification (Chen et al. 2001; Kenny 1979; Rindskopf 1984).

In this current study, an inspection of higher order model outputs in all three samples revealed negative residual variance on the fluid reasoning factor and a correlation greater than one between the second-order ‘g’ and the fluid reasoning factor. Further, the output produced a warning describing the covariance matrix as not positive definite and an ‘undefined’ fluid reasoning factor. Inspection of factor correlations found several factor correlations above 0.9, approaching 1, suggesting multicollinearity resulting in unstable parameter estimates when running a higher order model. As such, we concluded the higher order model was empirically underidentified and was not investigated further in this study.

However, statistical underidentification does not invalidate any theoretical higher order ‘g’ models or use of the FSIQ (Bowden et al. 2008a; Rindskopf and Rose 1988; Wilson et al. 2023c); instead, it only illustrates that the data conditions do not provide an optimal opportunity to estimate and evaluate higher order models. Importantly, if measurement invariance is established in any first-order model, then measurement invariance is implied to hold for any second-order factor or summary score that is based on the same first-order factor pattern (Widaman and Reise 1997).

Further, bifactor models, where all subtests additionally load onto an uncorrelated (orthogonal) higher order ‘g’ factor, were not explored as bifactor models in these dataalso have the issue of empirical underidentification leading to statistical estimation problems. Such estimation problems typically require arbitrary fixing of parameter values to obtain admissible solutions (Canivez et al. 2017, 2020, 2021; Decker 2020; Fenollar-Cortés and Watkins 2019; Markon 2019; Wilson et al. 2023c). Ideally, well-identified factor analytic models require at least three indicators per factor for the identification of higher order models. However, additional indicators would necessitate the development of additional subtests which load onto the relevant factor, with the consideration that testing time constraints will often make higher order model specification impractical (Bowden et al. 2008b; Rindskopf and Rose 1988). Further, both higher order and bifactor models have been shown to have the same pattern of relations as the first-order models between subtests and factors using the WISC-V US standardization sample (Reynolds and Keith 2017).

Despite these statistical limitations, researchers have reported second-order and bifactor models across some of the different versions of the WISC-V. However, in one example, the researchers failed to describe how five-factor, bifactor models with only two indicators loading onto a first-order factor were able to achieve convergence, making replication difficult (Lecerf and Canivez 2018). Alternatively, researchers have reported arbitrarily constraining to equality parameter estimates for factors with only two indicators per factor to achieve the identification of five-factor bifactor models (Canivez et al. 2017, 2020, 2021; Fenollar-Cortés and Watkins 2019). For example, when investigating the construct validity of the WISC-V US, the researchers imposed equality constraints to achieve convergence with five-factor *bifactor* models where “Some first-order factors were underidentified because they were measured by only two subtests. In those CFA, the two subtests were constrained to equality before estimating bifactor models to ensure identification” (Canivez et al. 2017, p. 461). Paradoxically, on the next page in the same article, an equality constraint to facilitate the convergence of five-factor *higher order models* was described as follows “this ‘only masks the underlying problem’ (Hair, Anderson, Tatham, and Black, 1998, p. 610) indicating that these models ‘should not be trusted’ (Kline 2016, p. 237). Accordingly, neither fit indices nor loadings for these models are reported” (Canivez et al. 2017, p. 462). In other words, the very authors reporting bifactor models of WISC-V data acknowledge that the estimation problems produce models that should not be trusted to be good solutions. In addition, the above studies all fail to test whether simpler, first-order, and identified models without post hoc ‘fixes’, provided good fit to the data.

Further, an exploratory factor analysis (EFA) was not undertaken in this current research. Firstly, CFA provides many advantages over EFA, such as the ability to undertake significance testing between competing models (Brown 2015; Gorsuch 2003). Additionally, CFA uses previous research and theory to apply theory-based solutions (Gorsuch 2003). Further, CFA offers more flexibility over EFA and facilitates the investigation of a much greater variety of models (Widaman 2012). Other advantages of CFA over EFA include the ability to test more parsimonious solutions and, importantly for this current study the ability to evaluate the equivalence of measurement models across groups (Brown 2015). Lastly, with respect to the replication crisis in psychological research, CFA allows for the direct comparison of different models, whereas EFA does not.

Factorial invariance analysis:

Once the first-order baseline model was established across French, Spanish, and US samples, a multigroup CFA was then used to test for factorial invariance. First, the French and US samples were compared, followed by the Spanish and US, and, lastly, the French and Spanish. We used the increasingly restrictive hierarchical approach to factorial invariance, whereby we started with an unconstrained model, other than holding the pattern of factor loadings identical as a test of configural invariance (Bontempo and Hofer 2007; Meredith and Teresi 2006; Widaman and Reise 1997). If configural invariance was established, we added the constraint of equal factor loadings as a test of weak invariance. If weak invariance was found, we added the constraint of equality of intercepts as a test of strong invariance. Lastly, if strong invariance was concluded, we tested for strict invariance by additionally holding the indicator residuals to equality across samples. Configural invariance was supported by fit indices showing a CFI, TLI, and Gamma-hat greater than 0.950 and an SRMR of less than 0.080 (Hu and Bentler 1999; Marsh et al. 2004). Evidence for weak, strong, and strict invariance would be supported by changes in CFI or TLI of not greater than 0.010, change in RMSEA of less than or equal to 0.015, or changes in SRMR less than or equal to 0.030 (Chen 2007; Cheung and Rensvold 2002; French and Finch 2006). However, poorer measurement quality, for example, the magnitude of the factor loadings, has been shown to lead to worse data-model fit and, as such, the quality of measurement was also considered when testing invariance across groups (Kang et al. 2016). Further, strict factorial invariance, which assumes equivalent residual variances across groups, may be overly restrictive and is unnecessary for construct generalization (Horn and McArdle 1992). Next, structural invariance was investigated across the three pair-wise comparisons. Additional constraints were placed on the strict invariance model, first, equality of factor variances; second, equality of factor variances and factor covariances; and, last, equality of latent means (Widaman and Reise 1997). Loss of model fit was compared to the strict invariance model using the same criteria for the assessment of factorial invariance.

## 3. Results

### 3.1. Confirmatory Factor Analysis Baseline Model Estimation

The results of the baseline model are shown in Table 3. The French, Spanish, and US samples were investigated independently. The one-factor model, whereby all 15 subtests loaded onto a Spearman’s ‘g’ factor, used as a baseline for more complex models, did not fit the data well in all three samples.

Next, the four-factor model was investigated and displayed improved model fit for all three samples compared to the one-factor model. Further, the fit indices showed that the four-factor model was a good fit to the data.

**Figure 1:**
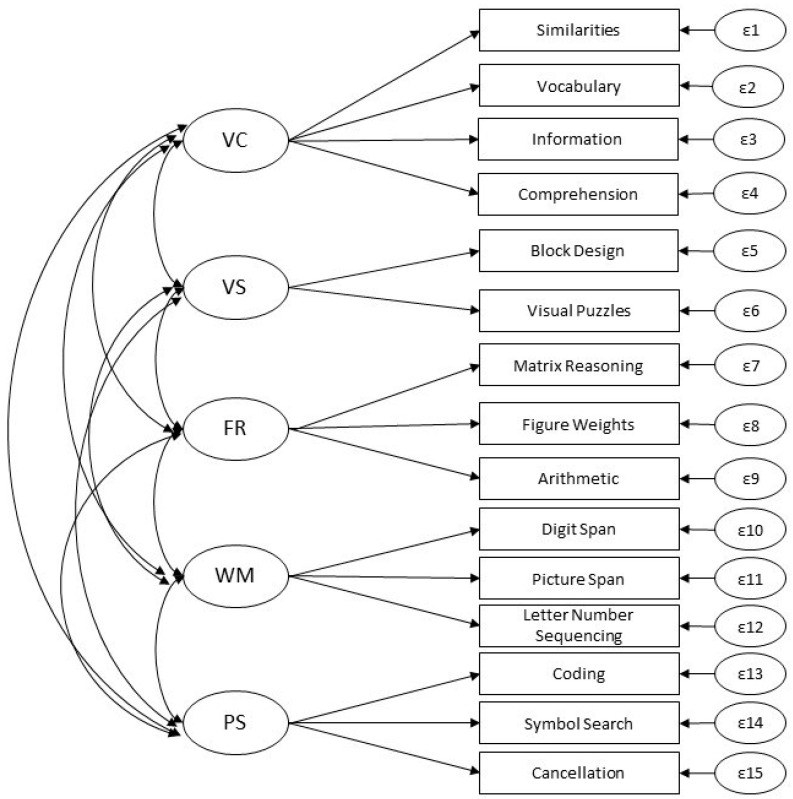
WISC-V European Scoring Factor Structure used as the Baseline Model. (Note. This first-order five-factor model was the best-fitting model in all samples independently and was the basis for testing of measurement invariance. ε = unique variances of the indicators. VC = Verbal Comprehension (Gc), VS = Visual Spatial (Gv), FR = Fluid Reasoning (Gf), WM = Working Memory (Gwm), PS = Processing Speed (Gs)).

Lastly, the published five-factor scoring model was compared for all three normative samples. The five-factor model displayed significantly improved fit compared to the four-factor model in all comparisons. Further, the evaluation of fit indices showed that the five-factor scoring model provides an excellent fit to the data in the French, Spanish, and US samples; for example, the CFIs were 0.980, 0.986, and 0.987, respectively. Examination of local fit revealed no evidence of indicator misfit. Thus, the first-order five-factor published scoring model (see Figure 1) was chosen as the baseline model for tests of factorial invariance across the three samples.

### 3.2. Measurement and Structural Invariance across France and the US

Results of the factorial invariance analysis across France and the US are presented in Table 4. For all tests of factorial invariance, the French sample was the reference sample. Configural invariance, where only the equality of the pattern of the five-factor model is held across samples, was first tested. The configural invariance model displayed excellent fit with a CFI of 0.985 and an RMSEA of 0.053, allowing for further restrictive tests of measurement invariance.

Next, weak invariance was tested by additionally holding the factor loadings to equality in both groups. The weak invariance model displayed no substantial loss of fit compared to the configural model across the French and US samples. Next, the strong invariance model also held the intercepts to equality across samples. Inspection of change in model fit showed a change of fit below the recommended cut off as evidence of strong invariance. As a final test of factorial invariance, strict invariance was investigated across the French and US samples by additionally holding the indicator residuals to equality. The results of the analysis showed no appreciable loss of fit compared to the strong invariance model with a change in RMSEA less than 0.001. Thus, it was concluded that the WISC-V displays strict factorial invariance across the French and US standardization samples.

Next, the structural components were analyzed for invariance across the French and US samples. First, using the strict invariance model, the factor variances were held to equality in both groups. The equality of variances model displayed no appreciable loss of fit compared to the strict invariance model. Additionally, equality of factor covariances was added to the equality of variances model. The change in model fit was again compared to the strict invariance model, with the results again suggestive of no loss of it. Finally, the equality of latent factor means was tested on the strict invariance model across French and US samples. No substantial loss of fit was found, providing evidence of the equality of latent means across the French and US standardization samples.

### 3.3. Factorial and Structural Invariance across Spain and the US

The same stepwise, increasingly restrictive hierarchical approach described above was used to assess factorial and structural invariance across the Spanish and US samples. The results of the invariance models are shown in Table 5. The configural invariance model displayed excellent fit with a CFI of 0.986 and RMSEA of 0.050. Next, an inspection of the change of fit across CFI, SRMR, and RMSEA indicated no substantial loss of fit for tests of weak, strong, and strict invariance. Thus, it was concluded that WISC-V displayed strict factorial invariance across Spanish and US standardization samples. Further, the equality of variances, equality of covariances, and equality of means models all showed no appreciable change in model fit compared to the strict invariance model.

### 3.4. Factorial and Structural Invariance across France and Spain

Lastly, the same approach to factorial and structural invariance was tested using the French and Spanish samples. The results of the invariance models are presented in Table 6. Again, the configural invariance model displayed excellent fit, with a CFI of 0.983 and RMSEA of 0.054. Next, an evaluation of the change in model fit was undertaken for the weak invariance, strong invariance, and strict invariance models. The results showed no discernible loss of fit, suggesting that the WISC-V displays strict factorial invariance across the French and Spanish standardization samples. Next, tests of structural invariance were tested compared to the strict invariance model. The results show that the French and Spanish samples displayed equality of factor variances and covariances. However, there was a significant loss of fit when the latent factor means were held to equality across samples. The results, therefore, suggest that in the French and Spanish standardization sample, latent factor means lack invariance.

**Figure A1:**
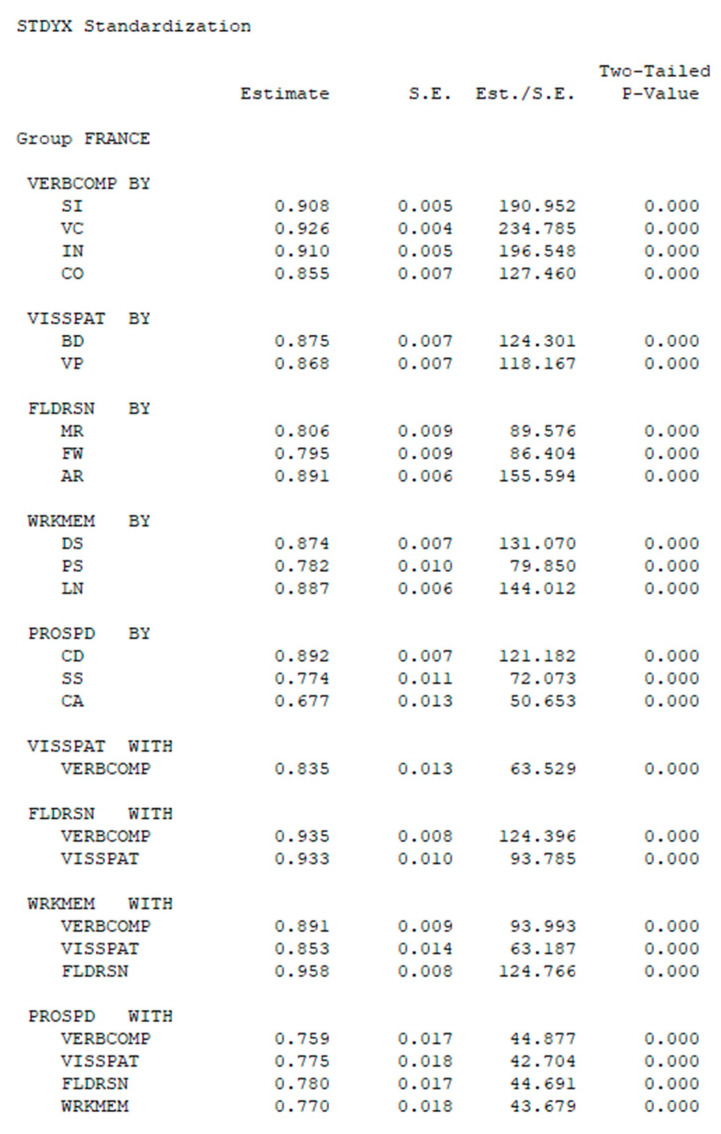
France Standardized Mplus Output of Strict Invariance Model between the WISC-V France and the US.

**Figure A2:**
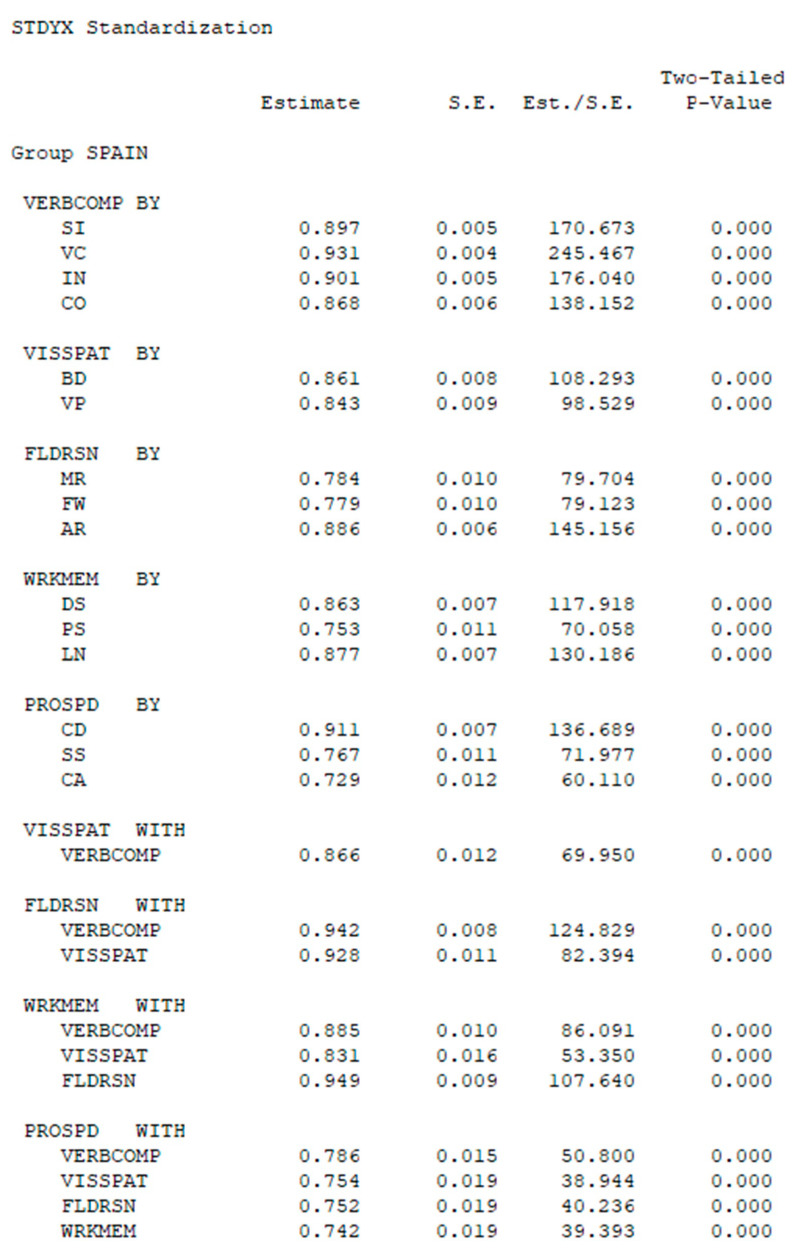
Spain Standardized Mplus Output of Strict Invariance Model between the WISC-V Spain and the US.

**Figure A3:**
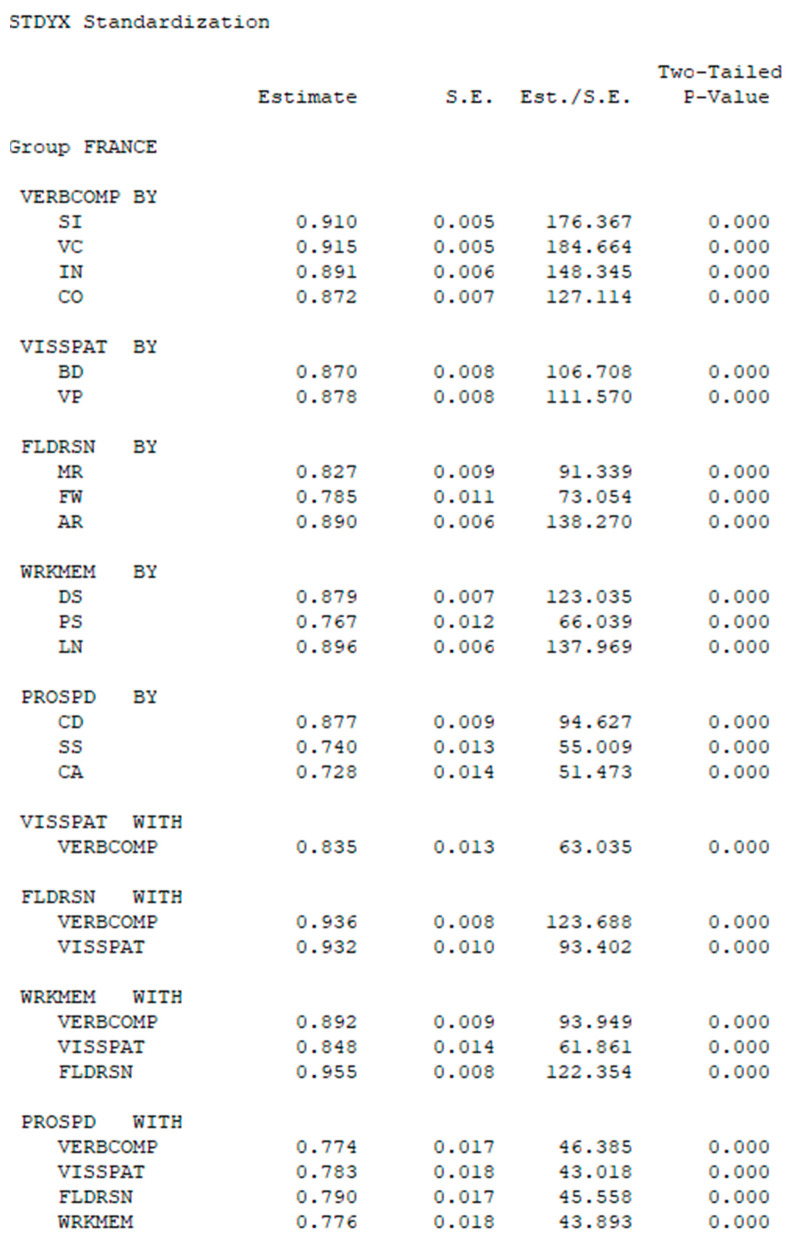
France Standardized Mplus Output of Strict Invariance Model between the WISC-V France and Spain.

Standardized parameter estimates of the three strict invariance analyses are available in the appendices, see Figure A1, Figure A2 and Figure A3.

### 3.5. Latent Means Comparisons

Establishing strong factorial invariance across all three pair-wise evaluations allowed for a statistically meaningful comparison of latent means across the French, Spanish, and US samples. As there were multiple comparisons, we applied the Bonferroni correction using a nominal alpha of 0.05, resulting in an adjusted alpha of 0.01. The French and US samples were again compared first and the results are presented in Table 7. The latent mean values were taken from the standardized output of the strict invariance model, with the French means set to zero and factor variances set to one in both samples. The results show small yet significant differences in the VC, FR, WM, and PS latent factor means, with the US performing higher across all four factors compared to the French sample. No significant difference was found in the VS factor.

Next, the Spanish and US sample latent factor means were compared. The results are shown in Table 8. The results were taken from the standardized output of the strict invariance model, with the Spanish means set to zero and variances set to one in both samples. The output shows a small but significant difference in the VC factor, with the Spanish sample performing higher. No significant differences were found across VS, FR, WM, or PS.

Lastly, the French and Spanish sample latent factor means were compared, and the results are presented in Table 9. Again, latent mean values were taken from the standardized output of the strict invariance model, with French sample latent means set to zero and variances set to one in both samples. The results show a medium and significant difference in the VC factor across French and Spanish samples, with the Spanish sample performing higher. This result supports the earlier finding of a lack of invariance across latent factor means across the French and Spanish samples. Small but significant differences were also found across WM and PS latent mean factors, again with the Spanish sample performing higher. No statistical differences were observed for the VS or FR factors across the French and Spanish samples.

## 4. Discussion

This study explored the generalizability of CHC constructs as measured by the WISC-V across French, Spanish, and US nationally representative samples. Invariance analysis supported the strict factorial invariance across (i) France and the US, (ii) Spain and the US, and (iii) France and Spain. The finding of strict factorial invariance provides strong evidence that the latent factors and the latent variable model, as measured by the WISC-V, measure the same psychological constructs which align with CHC theory across the three different language-speaking countries. Further, establishing factorial invariance of the WISC-V across France, Spain, and the US adheres to the guidelines set out by the International Test Commission on the use of translated and adapted tests (ITC 2017). Translating and adapting a test may change the meaning of an item or item difficulty, or cultural differences may cause an item to score differently across populations; however, the finding of strict metric invariance implies the constructs are equivalent across cultures and are estimated in equivalent scales.

Establishing factorial invariance across the three nationally representative samples also permits the comparison of latent mean differences (Meredith and Teresi 2006). When latent factor means were contrasted across the three samples, statistically significant differences were found of small and medium magnitude. Specifically, a medium and significant difference was found across the French and Spanish samples on the VC (comprehension knowledge; Gc) factor, with the French sample performing higher. Gc can be defined as the ability to understand and communicate culturally valued knowledge (Schneider and McGrew 2018). The language-based, culturally shared knowledge that is tested in Gc is learned through education and one’s environment (McGrew and Flanagan 1998; Weiss et al. 2019). Therefore, comparatively higher scores in comprehension knowledge may be the consequence of a relatively more cognitively enriching environment (e.g., parental education and income), which has been found to be a mediator of IQ, as well a relatively higher investment in formal education (Weiss and Saklofske 2020). Further, the difference may be attributable to the relatively higher government expenditure on education as a percentage of GDP between the two countries (UNESCO Institute for Statistics 2022).

However, findings of small latent mean differences on nationally representative samples are not uncommon (Bowden et al. 2008c; Wilson et al. 2023a, 2023c). As these results are based on nationally representative samples, any differences may be due to real differences across the three countries, differences in the sample recruitment methodology, or both. For example, a cross-cultural analysis using the WISC-V found that latent factor mean differences across countries aligned with country-level indicators of affluence and education (van de Vijver et al. 2019). The findings of the current study thus support the continued development of local normative data for high-stakes assessments, such as the WISC-V.

As demonstrated in a recent systematic review of the relevant cross-cultural literature (Wilson et al. 2023b), these results support and advance the research of Carroll (1993) by providing further evidence for the universality of the factorial constructs of the CHC model across a range of different language-speaking populations. The expanding literature supports the CHC model as a method of describing and understanding the structure of human cognitive ability and provides a common nomenclature and blueprint for future researchers and test developers (Jewsbury et al. 2016; McGrew 2009).

However, researchers exploring and expanding on the CHC framework should continue to apply best psychometric practice so as not to misconstrue model results. First, researchers are encouraged to use the more contemporary method of confirmatory factor analysis (CFA) over exploratory factor techniques which has been shown to provide imprecise and data-specific factor solutions leading to models that may be difficult to replicate. In contrast, a confirmatory approach has been shown to describe the true factor–indicator relationships and provide less biased results (Brown 2015; Gorsuch 2003; Jewsbury and Bowden 2017; Little et al. 1999; Widaman 2012). The continued use of EFA in studies of published tests is one of the key ingredients in the poor replicability of factor analytic studies, as noted by many previous authors (Brown 2015; Byrne 1989; Floyd and Widaman 1995; Henson and Roberts 2006; Kline 2016; Widaman 2012).

Further, researchers undertaking factor analysis should be aware of misspecifying models due to statistical underidentification, for example, by applying higher order models to a just-identified first-order model, which may lead to statistically inadmissible (though not theoretically inadmissible) results open to misinterpretation (Kline 2016; Rindskopf and Rose 1988). Moreover, to encourage further research of the hierarchical model of intelligence, it is important to note that demonstration of a fully measurement invariant first-order factor model implies invariance of a second-order ‘g’ or FSIQ factor model as long as the same transformations are applied by way of the so-*called ‘any rescaling factor’* (ARF, Widaman and Reise 1997) invariance. However, researchers have cautioned against the use of bifactor models in cognitive ability research unless theoretically and statistically justified (Decker 2020). The overwhelming factor analytic evidence on the Wechsler and other scales describes a CHC model with oblique, multidimensional, and highly correlated broad abilities (Chen et al. 2015; Flanagan et al. 2013; Schneider and McGrew 2018; van de Vijver et al. 2019; Weiss et al. 2013; Wilson et al. 2023c). Further, bifactor models often have model identification problems that cannot be resolved without arbitrary model restrictions, potentially leading to bifactor solutions with uncertain validity and an unknown degree of statistical bias (Canivez et al. 2017; Markon 2019; Reynolds and Keith 2017; Schneider and Newman 2015; Wilson et al. 2023c). In addition, bifactor models present prima facie interpretation problems for clinicians (which factor does a test score represent?) and have been used to perpetuate a contradiction (if the higher order, the general factor is the only sensible or more ‘reliable’ score on which to base an interpretation, why not show that a single-factor model fits the respective data sets best?).

A limitation of this research is that WISC-V was developed to measure five factors aligned with the CHC’s broad abilities (Weiss et al. 2019). Future researchers should extend the measurement invariance and construct validity of the CHC literature and explore the possible inclusions of other broad abilities. Additionally, further research is required to explore more diverse nationally representative samples as the present study analyzed representative samples from France, Spain, and the US, which are all Western or industrialized populations. The results leave open the question of whether or not the CHC constructs, as measured by the WISC-V, will generalize across non-Western or developing countries.

In conclusion, the factor analytic model underlying the WISC-V was demonstrated to be invariant across the French, Spanish, and US normative samples. As the WISC-V is aligned with the CHC structure of intelligence, the results provide further evidence of the generalizability of the CHC model across broad populations and allow for a common meaning and interpretation of cognitive ability test scores across those populations.

## Figures and Tables

**Table 1:** CHC Broad Abilities and WISC-V Factor Correspondence.

CHC Broad Ability	Corresponding WISC-V Factor	Broad Ability and Factor Definition
Comprehension Knowledge (Gc)	Verbal Comprehension (VC)	The ability to comprehend and communicate culturally valued knowledge
Visual Processing (Gv)	Visual Spatial (VS)	The ability to use mental imagery to perceive, discriminate, and manipulate mental images to solve problems
Fluid Reasoning (Gf)	Fluid Reasoning (FR)	The use of deliberate and controlled mental processes requiring focused attention to solve novel problems
Working Memory Capacity (Gwm)	Working Memory (WM)	The ability to maintain and manipulate information in active attention
Processing Speed (Gs)	Processing Speed (PS)	The ability to control attention to automatically, quickly, and fluently perform simple repetitive cognitive tasks

Note. CHC definitions adapted from Schneider and McGrew (2018).

**Table 2:** Demographic Characteristics of the French, Spanish, and US Samples.

Variable	France	Spain	US
Total *N*s	1049	1008	2200
Female/males; *n* (%)	532/517 (49.3/50.7)	500/508 (50.4/49.6)	1100/1100 (50.0/50.0)
Mean age in years (*SD*) range	11.2 (3.1) 6–16	11.3 (3.2) 6–16	11.5 (3.2) 6–16

Note. Standardization data from the Wechsler Intelligence Scale for Children-Fifth Edition (WISC-V). Copyright © 2014, 2015 NCS Pearson, Inc. Used with permission. All rights reserved.

**Table 3:** Goodness-of-Fit Statistics for the Baseline Model Estimation of the WISC-V French, Spanish, and US Samples.

Baseline Model	χ^2^	*df*	Δχ^2^	Δ*df*	CFI	TLI	Gamma-Hat	SRMR	RMSEA (Lower Bound, Upper Bound; 95%)	AIC	BIC
One factor											
France	1334.350 *	90			0.912	0.898	0.861	0.043	0.116 (0.111, 0.122)	93,910.184	94,132.232
Spain	1225.464 *	90			0.916	0.902	0.869	0.042	0.112 (0.107, 0.118)	90,618.389	90,839.283
US	2917.176 *	90			0.914	0.899	0.853	0.042	0.120 (0.116, 0.123)	199,978.585	200,234.853
Four factors											
France	431.911 *	84	902.439 **	6	0.975	0.964	0.956	0.027	0.064 (0.058, 0.069)	93,019.745	93,271.399
Spain	380.619 *	84	844.845 **	6	0.978	0.973	0.963	0.024	0.059 (0.053, 0.066)	89,785.545	90,035.891
US	714.006 *	84	2203.170 **	6	0.981	0.976	0.964	0.021	0.058 (0.055, 0.062)	197,787.414	198,077.851
Five factors											
France	357.634 *	80	74.277 **	4	0.980	0.974	0.965	0.025	0.058 (0.052, 0.064)	92,953.468	93,224.860
Spain	273.884 *	80	106.735 **	4	0.986	0.981	0.975	0.022	0.049 (0.043, 0.056)	89,686.809	89,956.791
US	517.620 *	80	196.386 **	4	0.987	0.982	0.974	0.019	0.050 (0.046, 0.054)	197,599.029	197,912.246

Note. CFI = comparative fit index; TLI = Tucker–Lewis index; SRMR = standardized root mean square residual; RMSEA = root mean square error of approximation, AIC = Akaike information criterion; BIC = Bayesian information criterion. * *p* < 0.05 for χ^2^ test. ** *p* < 0.05 for Δχ^2^ compared to the previous model. Standardization data are from the Wechsler Intelligence Scale for Children-Fifth Edition (WISC-V). Copyright ©2014, 2015 NCS Pearson, Inc. Used with permission. All rights reserved.

**Table 4:** Summary of Tests of Measurement Invariance between the WISC-V France and the US.

Invariance Model	χ^2^	*df*	Δχ^2^	Δ*df*	CFI	ΔCFI	TLI	Gamma-Hat	SRMR	ΔSRMR	RMSEA (Lower Bound, Upper Bound; 90%)	ΔRMSEA
Configural Invariance	875.255 *	160			0.985		0.980	0.943	0.021		0.053 (0.049, 0.056)	
Weak Invariance	1012.451 *	170	137.196 **	10	0.982	0.003	0.978	0.936	0.037	0.016	0.055 (0.052, 0.059)	0.002
Strong Invariance	1480.941 *	180	468.49 **	10	0.972	0.010	0.968	0.903	0.044	0.007	0.067 (0.064, 0.070)	0.012
Strict Invariance	1607.015 *	195	126.074 **	15	0.970	0.002	0.968	0.895	0.046	0.002	0.067 (0.064, 0.070)	<0.001
Equality of Variances	1648.829 *	200	41.814 **	5	0.969	0.001	0.968	0.893	0.054	0.008	0.067 (0.064, 0.070)	<0.001
Equality of Covariances	1752.929 *	210	145.914 **	15	0.967	0.003	0.967	0.885	0.054	0.008	0.068 (0.065, 0.070)	0.001
Equality of Means	1883.881 *	200	276.866	5	0.964	0.006	0.962	0.879	0.055	0.009	0.072 (0.069, 0.075)	0.005

Note. CFI = comparative fit index; Δ = change in, TLI = Tucker–Lewis index; SRMR = standardized root mean square residual; RMSEA = root mean square error of approximation. * *p* < 0.05 for χ^2^ test. ** *p* < 0.05 for Δχ^2^ compared to previous model. Standardization data are from the Wechsler Intelligence Scale for Children-Fifth Edition (WISC-V). Invariance models Equality of Variances, Equality of Variances and Covariances, and Equality of Means stepwise changes were compared to the Strict Invariance model. Copyright ©2014, 2015 NCS Pearson, Inc. Used with permission. All rights reserved.

**Table 5:** Summary of Tests of Measurement Invariance between the WISC-V Spain and the US.

Invariance Model	χ^2^	*df*	Δχ^2^	Δ*df*	CFI	ΔCFI	TLI	Gamma-Hat	SRMR	ΔSRMR	RMSEA (Lower Bound, Upper Bound; 90%)	ΔRMSEA
Configural Invariance	791.504 *	160			0.986		0.982	0.949	0.020		0.050 (0.046, 0.053)	
Weak Invariance	886.295 *	170	94.791 **	10	0.985	0.001	0.981	0.944	0.034	0.014	0.051 (0.048, 0.055)	0.001
Strong Invariance	1256.890 *	180	370.595 **	10	0.977	0.008	0.973	0.918	0.038	0.004	0.061 (0.058, 0.064)	0.010
Strict Invariance	1367.956 *	195	111.066 **	15	0.975	0.002	0.973	0.912	0.044	0.006	0.061 (0.058, 0.064)	<0.001
Equality of Variances	1389.969 *	200	22.013 **	5	0.974	0.001	0.973	0.910	0.061	0.017	0.061 (0.058, 0.064)	<0.001
Equality of Covariances	1467.929 *	210	99.973 **	15	0.973	0.002	0.973	0.906	0.061	0.017	0.061 (0.058, 0.064)	<0.001
Equality of Means	1540.774 *	200	172.818 **	5	0.971	0.004	0.970	0.899	0.048	0.004	0.065 (0.062, 0.068)	0.004

Note. CFI = comparative fit index; Δ = change in, TLI = Tucker–Lewis index; SRMR = standardized root mean square residual; RMSEA = root mean square error of approximation. * *p* < 0.05 for χ^2^ test. ** *p* < 0.05 for Δχ^2^ compared to previous model. Standardization data are from the Wechsler Intelligence Scale for Children-Fifth Edition (WISC-V). Invariance models Equality of Variances, Equality of Variances and Covariances, and Equality of Means stepwise changes were compared to the Strict Invariance model. Copyright ©2014, 2015 NCS Pearson, Inc. Used with permission. All rights reserved.

**Table 6:** Summary of Tests of Measurement Invariance between the WISC-V France and Spain.

Invariance Model	χ^2^	*df*	Δχ^2^	Δ*df*	CFI	ΔCFI	TLI	Gamma-Hat	SRMR	ΔSRMR	RMSEA (Lower Bound, Upper Bound; 90%)	ΔRMSEA
Configural Invariance	631.518 *	160			0.983		0.978	0.941	0.024		0.054 (0.050, 0.058)	
Weak Invariance	664.610 *	170	33.092 **	10	0.982	0.001	0.978	0.938	0.030	0.006	0.054 (0.049, 0.058)	<0.001
Strong Invariance	922.192 *	180	257.582 **	10	0.973	0.009	0.969	0.910	0.037	0.007	0.064 (0.060, 0.068)	0.010
Strict Invariance	1040.416 *	195	118.224 **	15	0.969	0.004	0.967	0.901	0.036	0.001	0.065 (0.062, 0.069)	0.001
Equality of Variances	1091.164 *	200	50.748 **	5	0.968	0.001	0.966	0.896	0.060	0.024	0.066 (0.062, 0.070)	0.001
Equality of Covariances	1185.723 *	210	145.307 **	15	0.965	0.004	0.965	0.885	0.061	0.025	0.068 (0.064, 0.071)	0.003
Equality of Means	1578.114 *	200	537.698 **	5	0.950	0.019	0.948	0.848	0.068	0.032	0.082 (0.079, 0.086)	0.016

Note. CFI = comparative fit index; Δ = change in, TLI = Tucker–Lewis index; SRMR = standardized root mean square residual; RMSEA = root mean square error of approximation. * *p* < 0.05 for χ^2^ test. ** *p* < 0.05 for Δχ^2^ compared to previous model. Standardization data are from the Wechsler Intelligence Scale for Children-Fifth Edition (WISC-V). Invariance models Equality of Variances, Equality of Variances and Covariances, and Equality of Means stepwise changes were compared to the Strict Invariance model. Copyright ©2014, 2015 NCS Pearson, Inc. Used with permission. All rights reserved.

**Table 7:** France and US Means of Latent Factors Scaled to the Unit of the French Sample.

Factor	*PE*	*SE*	Est./*SE*	*p*
Verbal Comprehension	0.354	0.037	9.498	<0.001
Visual Spatial	−0.061	0.041	−1.485	0.137
Fluid Reasoning	0.146	0.040	3.661	<0.001
Working Memory	0.162	0.041	3.960	<0.001
Processing Speed	0.137	0.037	3.649	<0.001

Note. The top row shows parameter estimates (PE) and standard error (SE), scaled in the unit of the French sample. Standardization data are from the Wechsler Intelligence Scale for Children-Fifth Edition (WISC-V). Copyright ©2014, 2015 NCS Pearson, Inc. Used with permission. All rights reserved.

**Table 8:** Spain and US Means of Latent Factors Scaled to the Unit of the Spanish Sample.

Factor	*PE*	*SE*	Est./*SE*	*p*
Verbal Comprehension	−0.183	0.037	−4.952	<0.001
Visual Spatial	0.036	0.039	0.927	0.354
Fluid Reasoning	0.069	0.039	1.777	0.076
Working Memory	0.014	0.038	0.378	0.705
Processing Speed	−0.044	0.040	−1.121	0.262

Note. The top row shows parameter estimates (PE) and standard error (SE), scaled in the unit of the Spanish sample. Standardization data are from the Wechsler Intelligence Scale for Children-Fifth Edition (WISC-V). Copyright ©2014, 2015 NCS Pearson, Inc. Used with permission. All rights reserved.

**Table 9:** France and Spain Means of Latent Factors Scaled to the Unit of the French Sample.

Factor	*PE*	*SE*	Est./*SE*	*p*
Verbal Comprehension	0.586	0.048	12.161	<0.001
Visual Spatial	−0.109	0.051	−2.142	0.032
Fluid Reasoning	0.081	0.049	1.659	0.097
Working Memory	0.164	0.051	3.209	0.001
Processing Speed	0.167	0.047	3.584	<0.001

Note. The top row shows parameter estimates (PE) and standard error (SE), scaled in the unit of the French sample. Standardization data are from the Wechsler Intelligence Scale for Children-Fifth Edition (WISC-V). Copyright ©2014, 2015 NCS Pearson, Inc. Used with permission. All rights reserved.

## Data Availability

Restrictions apply to the availability of these data. Data was obtained from Pearson Clinical Assessment, used with permission.
